# Distraction by emotion in early adolescence: affective facilitation and interference during the attentional blink

**DOI:** 10.3389/fpsyg.2013.00580

**Published:** 2013-09-03

**Authors:** Sabine Heim, April A. Benasich, Andreas Keil

**Affiliations:** ^1^Infancy Studies Laboratory, Center for Molecular and Behavioral Neuroscience, Rutgers, The State University of New JerseyNewark, NJ, USA; ^2^Psychophysiology Laboratory, Department of Psychology, Center for the Study of Emotion and Attention, University of FloridaGainesville, FL, USA

**Keywords:** attention capture, emotion distraction, temporal competition, dual task, rapid serial visual processing

## Abstract

This study examined the extent to which early adolescents (aged 10–13 years) differ from adults in their sensitivity to attention capture by affective stimuli during rapid processing. A rapid serial visual presentation paradigm (RSVP) was implemented as a dual task, requiring the report of two green target stimuli embedded in a stream of distractors. Known as the “attentional blink” (AB), task performance is typically impaired when the first and second targets (T1 and T2, respectively) are separated by at least one distractor and about 200 ms of time. Here we used written verbs of pleasant, neutral, and unpleasant content as T1 items, while affectively neutral exemplars served as T2 and distractor events. The temporal distance between T1 and T2 was manipulated to contain either one distractor (intertarget interval 232 ms) or five distractors (intertarget interval 696 ms). Students reported pleasant T1 words more accurately, compared to neutral and unpleasant words, indicating facilitation of appetitive content on performance during RSVP. Emotional relevance of T1 was at the expense of T2 accuracy: at an intertarget interval of 232 ms (i.e., during the AB period), identification of (neutral) T2 words was impaired when preceded by pleasant and unpleasant T1s. No interference across targets was observed, however, beyond the blink period, in which T1 and T2 were separated by 696 ms. Thus, emotionally relevant events capture and hold attentional resources, at the cost of attentive processing in subsequent episodes. Contrary to our findings in adults, these capture effects were most obvious when the available capacity was limited, i.e., during the critical interval of the AB. The findings are discussed in light of the use of alternative cognitive strategies as development proceeds beyond early adolescence into adulthood.

## Introduction

Children spend their lives in rich visual environments often characterized by rapidly changing patterns of audiovisual stimulation. With media exposure now dominating the majority of waking hours for many children and adolescents, sensory systems face the challenge of processing a daunting stream of information, often at high spatial and temporal density (Rideout and Hamel, 2006; Bohn and Short, 2009; Yang et al., 2013). Given the limited capacity of sensory systems, a mechanism for prioritization is needed that amplifies relevant stimuli at the cost of other, competing, information. Such competition is particularly obvious when multiple concurrent stimuli have relevance for the observer, such as when internal motive states (e.g., wanting to be entertained) collide with task goals set by an external standard (e.g., homework and assignments). In this vein, directing limited resources toward a task stimulus (such as a textbook) is challenged by concurrent stimuli associated with a competing motive (say a TV is on in the same room), leading to cost effects, referred to as distraction.

In the laboratory, numerous studies in adults have demonstrated that emotionally engaging words or pictures are particularly effective distractors (e.g., Ihssen and Keil, 2009). In one study, Calvo and Castillo (2005) found that adult participants required more time to decide whether a neutral word denotes a real or nonsense word, when 300 ms prior to this lexical decision task an extraneous threat word (e.g., kill, virus) occurred in foveal vision. Similarly, observers showed impaired visual motion detection when the motion stimulus was accompanied by emotionally engaging task-irrelevant picture distractors (Müller et al., 2008). These findings have been taken to indicate prioritized processing of significant information, for instance, stimuli linked to threat and reward (Bradley, 2009). Emotional distraction across time is not specific to threat stimuli, however. Ihssen et al. (2007) presented university students pleasant, unpleasant, and neutral pictures (the distractors) prior to a lexical decision task on neutral verbs vs. verb-like pseudowords (the targets). Regardless of the pleasure category (e.g., appetitive romance vs. aversive attack scenes) and the distractor-target interval (80 ms, 200 ms, 440 ms), affectively arousing images delayed reaction times to word stimuli. Similar effects have been observed in other tasks, such as the “emotional interrupt” paradigm, in which a visual target is both preceded and followed by the same distractor picture (e.g., Mitchell et al., 2006).

In adults, effects of emotional distraction on subsequent cognitive function persist across several hundreds of milliseconds (Ihssen et al., 2007; Müller et al., 2008), even when a masking stimulus separates the distractor from the task (Ihssen et al., 2007). Heim et al. (2013) recently demonstrated that 11- to 13-year-old students are already prioritizing emotional stimuli, even at long distractor-task intervals. In this study, students were asked to judge whether a string of letters (either a valid neutral verb or verb-like pseudoword) denoted a word or a non-word shortly after viewing extraneous colored pictures. Affectively intense pictures (pleasant and unpleasant) impaired processing of subsequent word targets, leading to response speed delays in lexical decision up to about 50 ms, when compared to neutral images. Such interference effects emerged irrespective of the temporal distance, inducing increased reaction times for targets presented 200 or 600 ms subsequent to the emotional distractor. Thus, for 11- to 13-year-olds, paralleling findings in adults, emotional cues capture and hold shared resources, which are subsequently diminished for processing the target event.

**Figure 1:**
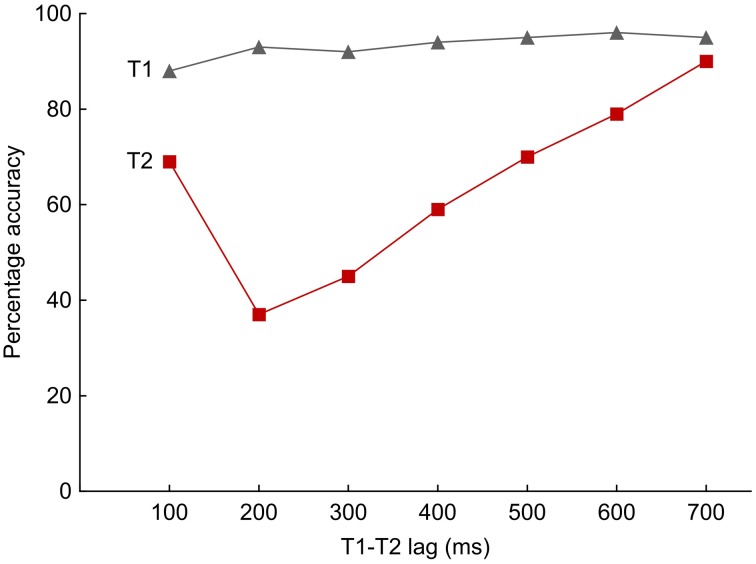
**A typical performance pattern in a dual-target rapid serial visual presentation task (i.e., an attentional blink task)**. The temporal distance between the two targets and the number of intervening distractors vary and result in different lag times. At a presentation rate of 10 Hz, a temporal separation of 100 ms contains no distractor, while at the 700-ms lag six distractors are embraced by the first and second targets (T1 and T2, respectively). The red curve displays the accurate T2 report given correct T1 identification as a function of lag times. Observers particularly often miss T2 if it falls in a window between 200 and 300 ms after T1, but dual-target report improves at longer lags. In many instances, superior accuracy translates to the earliest interval (100 ms), where T1 and T2 follow each other immediately. Single T1 report is shown in gray and is usually close to ceiling across lag times.

In the laboratory, capture effects are typically examined with stimulus arrays that are presented one at a time. This is different from many human-media interactions, which tend to involve sequences of stimuli, often presented at a rapid rate as is typical in computer games or on television. Therefore, research designs that require participants to cope with multiple attended objects competing for limited capacity are of particular interest. The rapid serial visual presentation (RSVP) paradigm is one such approach and allows researchers to examine the selection of predefined task-relevant items (the targets) from a stream of competing, irrelevant events (the distractors). Stimuli are delivered sequentially at high rates, typically 8–12 exemplars per second (see Raymond et al., 1992). Target items are often characterized by a specific feature, such as a certain color. When two targets are embedded within the temporal stream, then selectively attending to the first target (T1) tends to be associated with a transient impairment in detecting the second target (T2). This performance decrement is referred to as the “attentional blink” (AB) and is particularly evident when T1 and T2 are separated by at least one distractor and about 200 ms of time. When plotted as a function of intervening distractors, T2 report shows a hook-shaped accuracy profile in many studies, with accuracy at minimum with one or two intervening distractors, and relatively greater accuracy at zero or three and more intervening distractors (see Figure 1).

The neurocognitive mechanism underlying the AB effect has been debated and an extensive discussion of competing views is outside the scope of the present report. Considerable support exists for theoretical accounts of the AB that emphasize limited capacity and its allocation over time (e.g., Chun and Potter, 1995; Jolicoeur et al., 2006). Both empirical data and computational models have highlighted trade-off effects between T1 and T2, where over-allocation of attentional resources to T1 leads to T2 performance decrease. For instance, attenuated AB impairment is found when observers are prevented from over-attending T1, e.g., by listening to music (Olivers and Nieuwenhuis, 2005). Alternative perspectives have highlighted critical processes such as the temporal constraints of attention selection (Vul et al., 2008; Nieuwenstein et al., 2009), or differences in cognitive processing strategies (Shapiro et al., 2006; Wyble et al., 2009). Thus, a variety of factors affecting performance during rapid processing of multiple task items can be explored using AB-RSVP paradigms. In addition, since the blink phenomenon has been reliably demonstrated even with very simple stimulus materials, it provides a promising research design for developmental studies of attention.

In adults, the AB effect has often been used to examine costs and benefits associated with prioritizing emotional information: a series of studies has demonstrated that typically developing adults were more likely to report both targets in the critical blink period when emotionally intense or arousing stimuli served as T2 (e.g., Anderson and Phelps, 2001; Keil and Ihssen, 2004; Anderson, 2005; Keil et al., 2006). For instance, Keil and Ihssen (2004) found that arousing pleasant and unpleasant words (e.g., to fall in love, to rape) yielded about 15% higher identification scores than neutral exemplars (e.g., to label) during the AB window. In a study with electrophysiological recordings (Keil et al., 2006), this result was mirrored in rapid amplitude enhancement of the T2-evoked potential (120–270 ms after T2 onset) for pleasant and unpleasant targets specifically.

As a counterpart of the AB attenuation for affectively significant T2s, dual-target report tends to decline when distractor stimuli or T1 targets convey emotionally arousing information (Wang et al., 2012). A substantial body of research with the so-called emotional attentional blink paradigm (e.g., Most et al., 2005) has shown that even task-irrelevant stimuli (i.e., distractors) preceding a target during RSVP may elicit AB-like impairments in target report. The majority of studies with this paradigm have used pictures of faces or complex scenes and have converged to demonstrate relatively short-lived interference effects extending 100 s of milliseconds (McHugo et al., 2013). By contrast, studies manipulating the emotional content of an explicit (task-relevant) first target stimulus are relatively scarce (see e.g., Stein et al., 2009 for a discussion of this literature), and few studies have used visual words (e.g., Strauss et al., 2013). Of particular relevance to the present study, Ihssen and Keil (2009) examined both facilitation and interference effects induced by emotional content during the AB. Using words as stimuli, pleasant and unpleasant T1s were encoded with higher accuracy than neutral T1s. At the same time, T2 identification was impaired when following emotionally engaging T1 items, suggesting strong interference. Interference exerted by affective T1 words was temporally extended: across lags, T2s following pleasant or unpleasant T1s were less accurately reported than T2s subsequent to neutral T1s. This is consistent with other work manipulating the affective value of T1 words (Mathewson et al., 2008, Experiment 2). Thus, emotionally engaging leading distractors or first targets impair subsequent T2 report more so than neutral distractors and T1s. In addition, emotional distraction induced by affectively engaging words tends to be longer lasting than AB interference with neutral stimuli, and thus not specific to lag. This in turn can be taken to suggest that emotional distraction effects acting over time may not specifically reflect the same processes that lead to the AB phenomenon, but may reflect independent processes.

It is important in the context of the present study to consider developmental aspects of the AB effect. Heim et al. (2011) compared the behavioral accuracy of two groups of children in an AB task with non-linguistic symbols. Participants were asked to identify two green targets embedded in a white distractor stream. The temporal distance within the target doublet varied from no intervening distractor (lag 1 at 116 ms) up to seven intervening distractors (lag 8 at 928 ms). First-grade children (aged 6–7 years) showed a linear decrease in behavioral accuracy for the target pair with increasing temporal proximity of T1 and T2. Fifth and sixth graders (aged 10–11 years) exhibited a performance profile often observed in adult participants: they were able to quickly allocate their attention to two targets in a row, but this performance profile was accompanied by reduced report rates when T2 was preceded by an intermittent distractor (i.e., the AB phenomenon). Data from a large cross-sectional sample (Heim et al., under review) indicate that the most salient change in AB profiles occurs as a shift from a linear to a hook-shaped profile, between first and fourth graders. Around age 10–11 years, the blink pattern remains stable, with slight but constant increases in accuracy across temporal lags into later adolescence. Thus, in our present study, it is expected that early adolescents display pronounced performance deficits at short as compared to longer T1-T2 lags. As an additional variable, we manipulated the affective value of the target items (see below), increasing the amount of trials per lag by a factor of 3. Session duration in a sample of young participants, however, is particularly limited by motivational factors as well as fatigue. The present research therefore focuses on one early and one late lag, sampling the conditions shown to be associated with most inferior and superior accuracy, respectively.

The current study builds on these findings to examine possible emotion-induced facilitation and interference effects in a sample of 10- to 13-year-old typically developing students, by using a dual-target RSVP paradigm. This age group already shows a stable blink pattern (Heim et al., 2006, 2011; Heim and Keil, 2012) and is thus considered well-suited to test two related hypotheses: (1) It is expected that T1 identification is facilitated by emotionally arousing (pleasant and unpleasant) words in the AB task and that this facilitation is accompanied by corresponding T2 impairment. This first hypothesis would be supported if early adolescents show heightened sensitivity or enhanced allocation of resources to an emotionally engaging target, leading to error-prone processing of the trailing pertinent event. Such a pattern may be reflective of the interaction between a processing strategy (e.g., focusing on never missing T1) and the intrinsic saliency of the affective stimulus. Additional predictions can thus be made regarding the temporal dynamics of the anticipated interference effects: (2) If the blink phenomenon itself and the emotional interference effect are independent (Wang et al., 2012), as is often observed in AB studies with word stimuli conducted in adults (Mathewson et al., 2008; Ihssen and Keil, 2009), then a temporally extended pattern of T1 interference on T2 report would be expected, affecting early and late lag conditions. This hypothesis would be consistent with our previous findings in 11- to 13-year-olds (Heim et al., 2013), suggesting temporally sustained interference effects, possibly reflecting general prioritization of emotionally engaging distractors in attention, cognitive, and motor systems (Heim and Keil, 2012). By contrast, a short-lived interference phenomenon and hence a failure to support hypothesis 2 implies an attention-specific effect of emotional interference, such that affective word content of T1 selectively heightens the AB impairment in young adolescents.

## Materials and methods

### Participants

Eighty-three students (37 males) between the ages of 10 and 13 years (*M* = 11.73 years, *SD* = 0.72) volunteered in the present study. At the time of testing, participants had completed fifth and sixth grades of German secondary schools located in the federal states of Baden-Württemberg and Bavaria. The present sample included a cross-section of both city and suburban families, reflecting the socioeconomic status distribution of the particular area. German was the primary language for each participant. Students with a history of specific language impairment and reading disability, or suffering from any neurological disease (e.g., brain injury) and/or psychiatric condition (e.g., affective spectrum disorder) were not included in this study. Participants did not take any psychoactive medication and had normal or corrected-to-normal vision.

Because stimuli in the experimental paradigm (see section Attentional blink task) were delivered very rapidly, only seizure-free students with a negative first-degree family history of epilepsy were examined. Participants were also evaluated in terms of their basic reading skills to ensure sufficient word identification in the task. To this end, we administered the Salzburg Reading Screening for Grade Levels 5–8 (Auer et al., 2005), which was designed to assess accurate and fluent reading by judging the content of syntactically and semantically simple sentences as either true or false. The number of correct judgments within a preset time of 3 min was transformed to a grade-level based reading quotient with a mean of 100 and a standard deviation of 15. All participants scored in the average to above-average range (90–150), with a mean reading quotient of 110.90 (*SD* = 12.12) across the whole sample.

### Study protocol

The study protocol was approved by the ethics committee of the University of Konstanz and adhered to the Declaration of Helsinki. Written informed consent was obtained from the parents of the students prior to the testing session; students gave their verbal assent. Assessment took place one participant at a time in a quiet room provided by the schools. Students completed three measures, including the standardized reading test (see section Participants), the AB task, and post-experimental affective stimulus ratings. Including breaks, a typical session lasted 60 min with the AB task averaging approximately 30 min. Instead of compensating each participant at the end of the session, a financial bonus was given to the class fund for common student projects.

#### Attentional blink task

German verbs served as stimuli in the AB task. The T1 stimuli consisted of 20 pleasant arousing (e.g., to party, to win), 20 neutral (e.g., to measure, to operate), and 20 unpleasant arousing words (e.g., to fight, to poison). Another set of 20 neutral items constituted the T2s (e.g., to count, to name). The majority of these verbs were compiled based on our unpublished ratings by 49–180 school-age students (9–13 years old) on hedonic valence (1 = highly unpleasant to 9 = highly pleasant) and arousal (1 = low arousal to 9 = high arousal). Mean valence and arousal scores were 5.43 (*SD* = 0.27) and 3.95 (*SD* = 0.40) for the total number of neutral target words (*n* = 40), 7.96 (*SD* = 0.53) and 6.87 (*SD* = 0.84) for pleasant words (*n* = 17), and 1.90 (*SD* = 0.66) and 6.06 (*SD* = 0.36) for unpleasant exemplars (*n* = 17). The remaining emotional words were selected from a series of rating studies in a total of 215 college-age students (Ihssen and Keil, 2009), resulting in mean valence and arousal values of 7.85 (*SD* = 0.44) and 6.16 (*SD* = 1.33) for the pleasant (*n* = 3) category, and 1.44 (*SD* = 0.27) and 7.71 (*SD* = 0.66) for the unpleasant category (*n* = 3). A subset of 30 words, randomly extracted from the entire target pool, was evaluated by the present sample upon completion of the AB task (see section Post-experimental affective word ratings). The complete set of target words is presented in the Appendix; items belonging to the post-experimental rating subset are marked.

The pleasant, neutral, and unpleasant target lists were matched for phonological word length defined as the number of syllables, *F*_(3, 76)_ = 0.34, *p* < 0.795, with an average of 2.38 syllables (*SD* = 0.49) across the lists. This variable has been shown to modulate the magnitude of the blink effect (Olson et al., 2001). All of these words were well-represented in prevailing fifth-grade reading and text books. Thus, we used stimuli that the participants knew well. Knowledge of the target items was also tested by having each student read aloud the series of verbs once in a fluent manner at the beginning of the experimental session.^1^ All students successfully completed this initial test. An additional 60 neutral verbs served as the distractor set. Having an average number of 9.65 letters (*SD* = 2.15) the distractors were visually longer than the target events (*M* = 7.36, *SD* = 1.48; *p* < 0.001), which enabled sufficient target-by-distractor masking (Anderson and Phelps, 2001).

**Figure 2:**
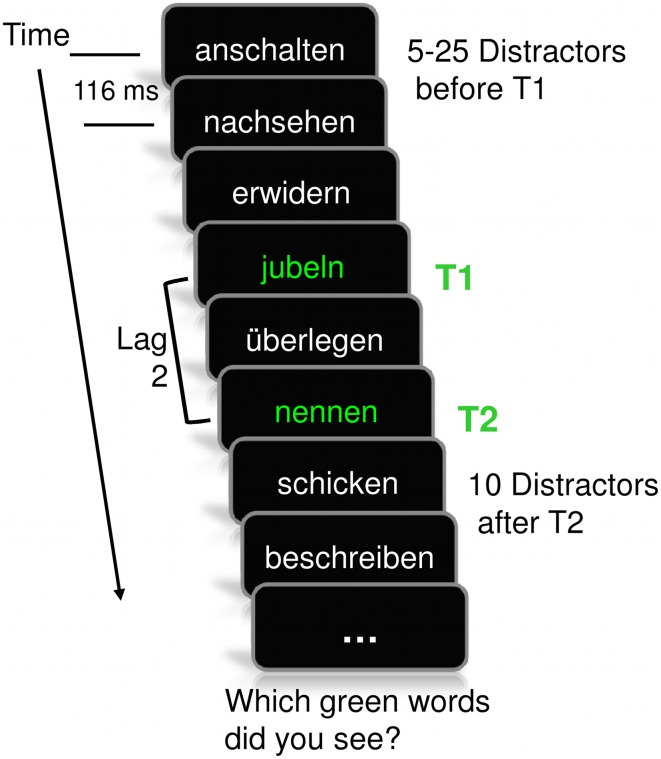
**Schematic of the rapid serial visual presentation paradigm, implemented as an attentional blink task**. Students were asked to identify two target words occurring in green amidst a series of white words (distractors). The first target (T1) varied in affective value (arousing pleasant, neutral, arousing unpleasant), while the second target (T2) and the distractors were always neutral in content. Each trial included a baseline period of distractors, varying in number, before T1 was displayed. T2 was followed by another sequence of distractors. The temporal distance within the target doublet was 232 ms (one intervening distractor) or 696 ms (five intervening distractors), reflecting lags 2 and 6, respectively. The present example illustrates a trial with an engaging pleasant T1 in the lag 2 condition. Note: anschalten, to turn on; nachsehen, to check back; erwidern, to respond; jubeln, to cheer; überlegen, to reason; nennen, to name; schicken, to send; beschreiben, to describe.

Target and distractor stimuli appeared centrally on a computer screen with a retrace rate of 60 Hz, at an average distance of 50 cm from the observer. A script written using Presentation software (Neurobehavioral Systems, Inc., Albany, CA, USA) controlled stimulus delivery. Targets appeared in bright green and distractors in white color, each in lowercase characters, using 30-point Times New Roman font against a black background. Each word on the screen subtended a vertical visual angle of 0.86°. Target words had a luminance of approximately 24.9 cd/m^2^. Each stimulus in the stream was displayed for 50 ms, followed by a blank screen for 66 ms, which resulted in a rapid presentation rate of 8.7 items per second. A trial started with a randomized number of 5–25 distractors to avoid anticipation of T1 occurrence. The T1 verb (pleasant, neutral, or unpleasant in content) was followed by either one or five distractors until a neutral T2 verb appeared. These T1-T2 intervals represent lag 2 with a stimulus-onset asynchrony (SOA) of 232 ms and lag 6 with an SOA of 696 ms, respectively. The T2 was always succeeded by 10 distractors. A schematic of an example trial is shown in Figure 2.

There were 20 trials for each combination of lag (2 vs. 6) and T1 category (pleasant, neutral, and unpleasant), resulting in a total of 120 trials. A break was scheduled after 60 trials to ensure students remained focused. The sequence of conditions was pseudo-randomized to control for immediate repetitions of the same target words. Participants were invited to monitor the stimulus stream for two green words and to report their identity after each trial. Responses were delivered orally and recorded by the experimenter. They were instructed to guess when unsure about the word; no feedback was provided. After response completion, participants initiated the next trial using the space bar. The AB task included five practice trials prior to testing; practice verbs were different from the test exemplars.

#### Post-experimental affective word ratings

In order to validate that the target words in the AB task were experienced according to their affective category, all participants engaged in post-experimental ratings. To keep the length of the testing session within reasonable time limits for each student, 30 words from the entire T1 (10 pleasant, 10 unpleasant, 5 neutral verbs) and T2 lists (5 neutral verbs) were randomly selected. As shown in the Appendix, the obtained stimulus selection was not characterized by extreme pleasant and unpleasant exemplars, which may have biased the affective evaluations. Participants completed subjective ratings regarding hedonic valence and arousal on this subset of target items by using a paper-and-pencil version of the Self-Assessment Manikin (SAM; Bradley and Lang, 1994). Whereas judgments of hedonic valence indicate whether a stimulus is perceived as pleasant, neutral, or unpleasant, judgments of arousal reflect the intensity of motivational activation (Bradley and Lang, 1994). A figure depicting the valence dimension of the SAM ranges from a big smiling, happy manikin to a frowning, unhappy manikin. Words such as very happy, pleased, or good and very unhappy, sad, afraid, or bad were used to make the endpoints accessible to the students. A jumping, wide-eyed manikin with bursts in its belly at one extreme and a calm, sleeping manikin at the other characterize the arousal dimension. Expressions such as excited, nervous, butterflies in one's tummy, active, or wide-awake and extremely calm, relaxed, bored, or sleepy gave students an understanding of the extremes. The unlabeled dimensions include five manikins, each separated by a small box. Marks could be made on the manikin or the box, resulting in a 9-point assessment scale per dimension. Participants were instructed to rate a given word first on the valence dimension, and then with respect to its arousal. Practice on two sample verbs not from the experimental set ensured that each student understood the assessment procedure (for using the SAM successfully in children and adolescents see McManis et al., 2001; Müller et al., 2004; Heim et al., 2013).

#### Data analysis

In the AB task, target identification performance was expressed as the percentage of correct responses for each of the six experimental conditions (2 SOAs × 3 affective T1 categories). T2 report was considered correct only on trials with accurate T1 identification (T2|T1 accuracy). This is generally assumed to emphasize specific effects of limited resources across the two targets (Raymond et al., 1992). Subsequently, separate *F* values for T1 and conditional T2 responses were calculated using repeated measures analyses of variance (ANOVAs) crossing the within-participants factors Lag (2; 2 = 232-ms SOA, 6 = 696-ms SOA) and Affective T1 Category (3; pleasant, neutral, unpleasant). Where appropriate, contrast analyses were used to follow up significant ANOVA results.

Regarding SAM ratings, mean valence and arousal values for each affective category and participant were determined and submitted to separate repeated measures ANOVAs, with Affective Category (3; pleasant, neutral, unpleasant) as the within-participants factor. Contrast analyses were planned to further investigate significant main effects. For all analyses, effects were deemed significant when *p* < 0.05.

## Results

### Performance in the attentional blink task

**Figure 3:**
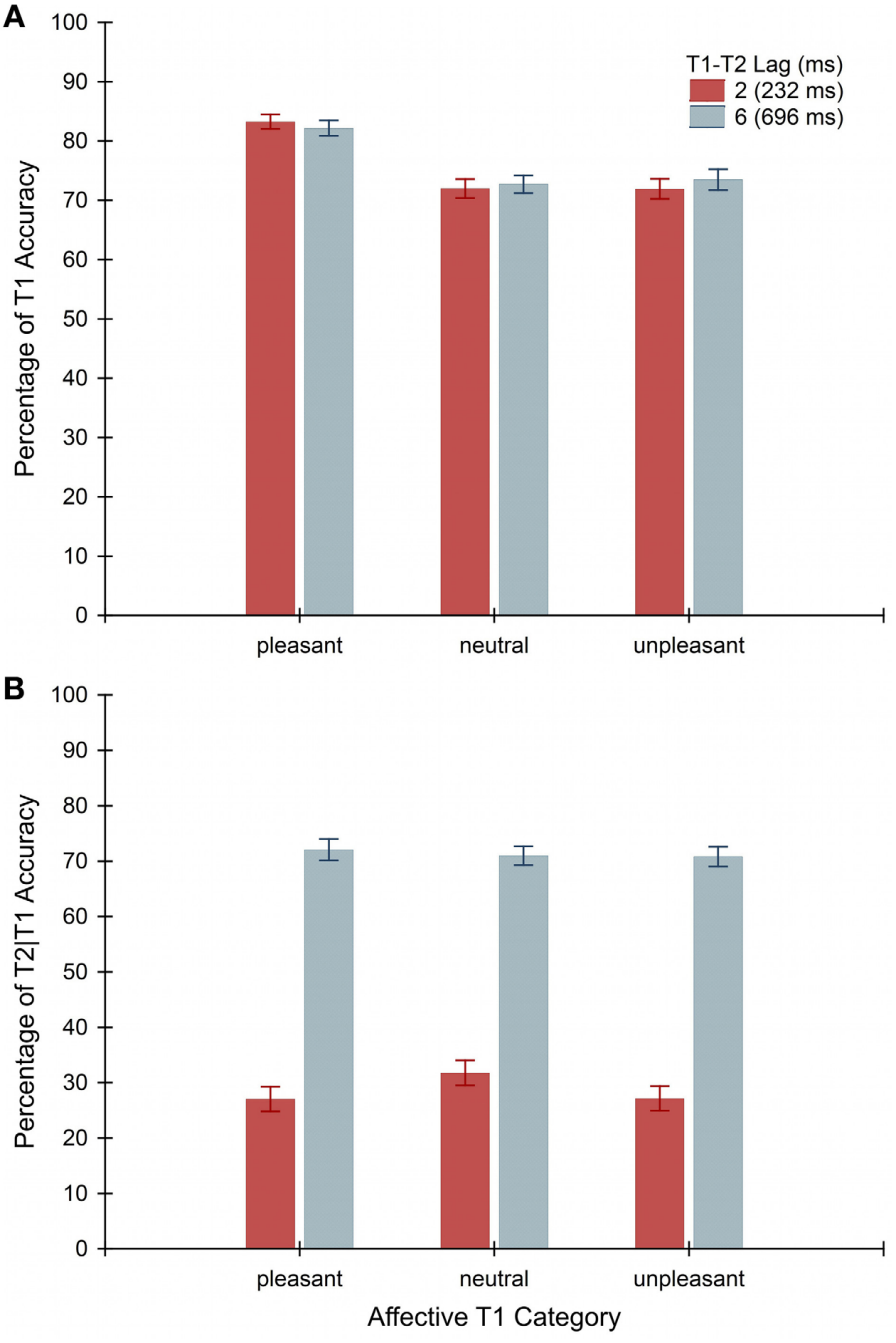
**Mean behavioral accuracy (*n* = 83 students) in the attentional blink task as a function of affective T1 category at the 232-ms (red bars) and 696-ms (blue bars) interval between the first and second targets (T1-T2 lag)**. Error bars indicate standard errors of means. Plot **(A)** illustrates the percentage of correctly identified T1 items. Plot **(B)** displays the percentage of accurate T2 report given T1 identification.

ANOVA on T1 accuracy revealed a significant main effect of Affective T1 Category, *F*_(2, 164)_ = 47.48, *p* < 0.001, indicating facilitated identification of appetitive content across lags (see Figure 3A). Contrast analyses confirmed significantly superior performance for pleasant (*M* = 82.71, SEM = 1.14) compared to neutral (*M* = 72.35, SEM = 1.43), *F*_(1, 82)_ = 96.44, *p* < 0.001, and unpleasant T1 words (*M* = 72.71, SEM = 1.63), *F*_(1, 82)_ = 72.31, *p* < 0.001. Report rates on neutral and unpleasant exemplars did not differ. The ANOVA yielded neither a main effect of Lag nor a Lag by Affective T1 Category interaction for T1s.

In terms of T2 accuracy, we first established whether the data were consistent with a transient impairment in dual-target performance during the critical blink interval. Supporting an AB effect, conditional T2 report depended on Lag with significantly lower accuracy at lag 2 (*M* = 28.66, SEM = 2.06) than lag 6 (*M* = 71.28, SEM = 1.56), *F*_(1, 82)_ = 676.14, *p* < 0.001. This main effect in the ANOVA was affected by a significant Lag × Affective T1 Category interaction, *F*_(2, 164)_ = 4.21, *p* < 0.017. Follow-up contrasts revealed that identification of T2 words was systematically impaired when preceded by pleasant and unpleasant relative to neutral T1s in the lag-2 condition, *F*_(1, 82)_ = 11.41, *p* < 0.002 and *F*_(1, 82)_ = 7.77, *p* < 0.007, respectively. No influence of Affective T1 Category on T2 performance was observed in the later temporal lag 6 (see Figure 3B), which mirrored the non-significant main effect of this factor.

### Post-experimental affective word responses

**Figure 4:**
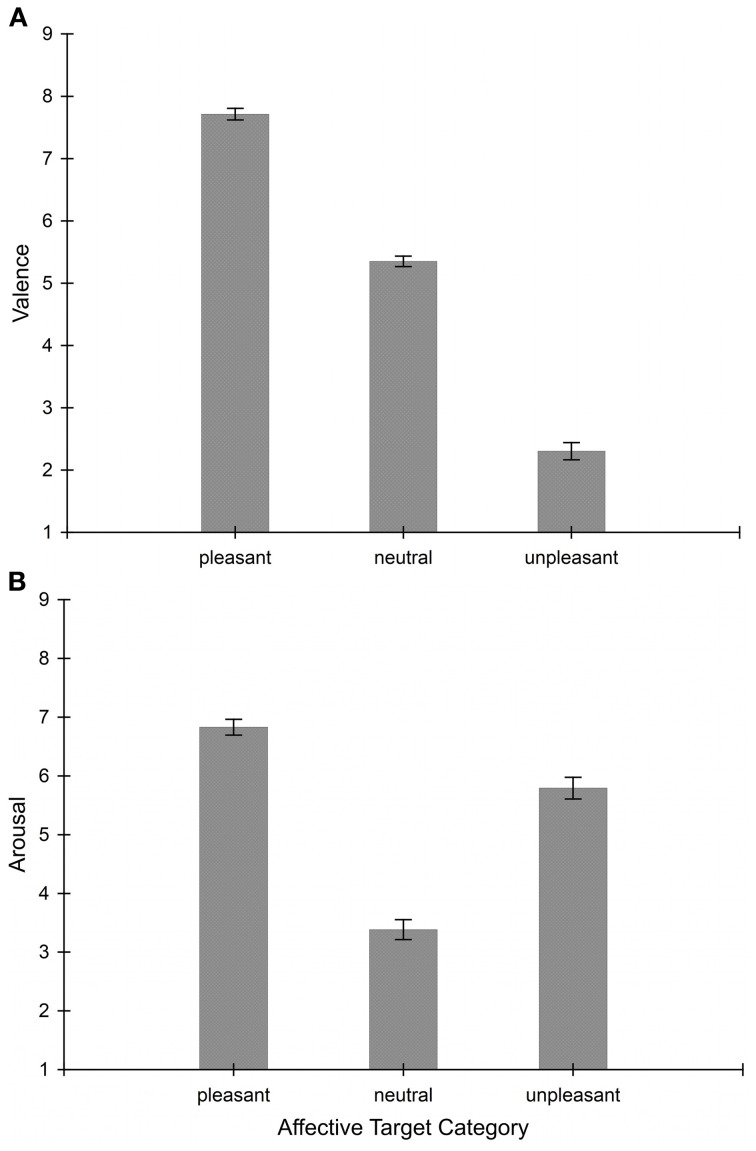
**Subjective ratings for 30 randomly selected words from the pleasant, neutral, and unpleasant target lists (10 from each affective category) used in the attentional blink task**. Values represent means of 83 students; error bars indicate standard errors of means. Plot **(A)** depicts valence scores rated on a scale of 1 = highly unpleasant to 9 = highly pleasant. Plot **(B)** shows ratings on the arousal dimension between the extremes 1 = low and 9 = high arousing.

Students rated the target contents significantly different in terms of hedonic valence, *F*_(2, 164)_ = 631.92, *p* < 0.001. Contrast analyses revealed that pleasant words were judged as more appetitive than neutral words, *F*_(1, 82)_ = 644.44, *p* < 0.001, and unpleasant words, *F*_(1, 82)_ = 814.65, *p* < 0.001. Furthermore, verbs of unpleasant content were considered less pleasurable than exemplars from the neutral category, *F*_(1, 82)_ = 367.48, *p* < 0.001 (see Figure 4A).

Students' ratings on the arousal dimension varied with affective word content as well, *F*_(2, 164)_ = 145.53, *p* < 0.001. Follow-up testing of the significant ANOVA result indicated that pleasant and unpleasant verbs were rated as more arousing than neutral verbs, *F*_(1, 82)_ = 371.70, *p* < 0.001 and *F*_(1, 82)_ = 111.61, *p* < 0.001, respectively. In addition, arousal scores for pleasant T1 items significantly exceeded those of the unpleasant type, *F*_(1, 82)_ = 23.95, *p* < 0.001 (see Figure 4B).

## Discussion

The present findings demonstrate that, in early adolescents, emotionally salient words quickly capture and hold significant portions of the limited capacity available during rapid serial processing. Supporting the blink phenomenon as typically found in this age group (Heim et al., 2006, 2011; Heim and Keil, 2012), successful report of the target doublet was impaired at short (232 ms) compared to late (696 ms) T1-T2 lags. As expected, emotional relevance of T1 was at the expense of T2 performance: at the 232-ms lag (i.e., during the AB period), identification of (neutral) T2 words was impaired when preceded by pleasant and unpleasant T1s. Contrary to previous work in adults (Ihssen and Keil, 2009), such interference did not translate to the late lag condition, in which T1 and T2 were separated by 696 ms. In summary, students in their early adolescence showed a T1 benefit based on pleasant word content, yet both pleasant and unpleasant words exerted significant interference on the T2 report accuracy. These distraction effects were only evident during the critical AB interval. Valence and arousal ratings of a randomly selected target sample support that our students perceived the words according to their affective value. The present findings give only partial support to our two related hypotheses: (1) Pleasant T1s were indeed characterized by superior report rates, however, this was not the case for unpleasant T1s as we had anticipated. (2) Interference of emotionally charged T1 words was evident in the early temporal lag, but did not extend into the late lag condition.

The overall pattern of these results is consistent with a growing literature using variants of the AB paradigm, in conjunction with emotional stimuli, to study distraction caused by the presence of task-irrelevant, but affectively engaging stimuli. For instance, a substantial body of research has relied on the emotional AB paradigm (Most et al., 2005), in which target stimuli embedded in an RSVP stream are preceded by task-irrelevant distractor items varying in affective content (McHugo et al., 2013). Converging with the present findings, this literature points to strong interference effects when leading distractors are emotionally engaging. In terms of time course, reports have repeatedly demonstrated that highly appetitive distractors, such as erotic pictures, tend to be extremely effective in inducing cost effects even at short lag intervals (Ciesielski et al., 2010). Unpleasant distractors also have been shown to exert substantial interference, particularly when observers display strong emotional reactivity in response to the distractor category, as is the case in anxiety disorders (Olatunji et al., 2011, 2013).

In the study described here, the *affective value of T1* was manipulated rather than varying the content of a preceding distractor. This aspect of the experimental design enables estimating benefits of emotional T1 content on first target processing. Testing hypothesis 1, we found superior report rates for pleasant T1 words, compared to neutral and unpleasant items. This is partly in contrast to prior work in adults (Ihssen and Keil, 2009), demonstrating that emotionally intense appetitive as well as aversive words facilitate T1 identification. Ratings of emotional arousal obtained for a subset of words, however, suggest that our school-age students perceived the appealing word material as more engaging than unpleasant exemplars. This is consistent with previous research, observing that adolescents (12–14 years of age) exhibited stronger coupling of emotional arousal and hedonic valence ratings in response to appetitive, compared to aversive pictures (McManis et al., 2001). It is plausible that such heightened sensitivity of appetitive motive systems translated into the rating differences and T1 report effects observed in our study. The potency of pleasant information for rapidly capturing attention has been noted in adult samples as well (Keil et al., 2002; Olatunji et al., 2011) and its developmental trajectory is deserving of future investigation.

The main finding regarding hypothesis 2 was pronounced early interference, characterized by impaired T2 report following emotionally salient, pleasant and unpleasant, T1 words. Contrary to previous data in adults (Ihssen and Keil, 2009), these cost effects were short-lived: no effects of T1 content on T2 report were found for the longer lag, when T2 succeeded T1 within 696 ms. This temporal pattern is unexpected to the extent that research with picture distractors in young adolescents of comparable age to our participants has pointed toward temporally sustained interference by a leading distractor (Heim et al., 2013): presenting emotionally engaging irrelevant pictures prior to a mask and a (target) lexical decision task resulted in prolonged response times across distractor-target SOAs of 200 and 600 ms, for pleasant and unpleasant distractors alike, paralleling work in adults (Ihssen et al., 2007). Overall, studies of emotional interference have shown considerable variability regarding the time course of these emotion-related impediments. Whereas work with implicit (task-irrelevant) leading emotional distractors tends to find short-lived interference, paradigms in which a first target is task-relevant and affectively engaging often revealed more sustained interference with subsequent processing. In terms of potential mechanisms for these different dynamics, electrophysiological studies have pointed to the fact that prolonged interference is observed when competition among stimuli persists beyond perception and attention systems. For instance, creating competition between irrelevant distractors and subsequent task items on the level of semantic content leads to sustained impairment accompanied by suppression in neural markers of semantic processing (Ihssen et al., 2007). In a similar fashion, prolonged interference is observed when conflicting response tendencies are established between an emotional distractor and a subsequent target (Ihssen and Keil, 2013). In the present context, these studies suggest that interference exerted by an emotionally arousing T1 word primarily affects perceptual and attentional processes in young adolescent observers.

As an alternative interpretation of the finding related to hypothesis 2, it is interesting to consider aspects of the task as well as of overall performance level. Sustained interference is often seen in adults when task requirements are challenging and report accuracy is low (Ihssen and Keil, 2009; Keil and Heim, 2009). Interference also tends to be particularly pronounced when using emotionally strong and complex distractors, such as colored pictures displaying highly arousing scenes (Schönwald and Müller, 2013) that trigger sustained processing (Bradley et al., 2012). In addition, it is well-established that AB performance is affected by the strategy adopted by a given observer to address the difficulty of reporting both targets (Shapiro et al., 2006; Martens and Wyble, 2010). Most importantly, trade-off effects between T1 and T2 are routinely reported when considering individual trials (Shapiro et al., 2006). In the present study, T1s were word stimuli, and as such not as effective in engaging emotional responses as pictures or movies (Keil et al., 2006). Furthermore, the leading distractor (T1) was a target event, inducing concurrent processing of affective value and task-relevance. Given these properties, sustained interference of affective T1 content is expected when the T2 task is particularly challenging, or when participants opt for a strategy that focuses on T1 at the cost of T2, heightening vulnerability of the T2 to interference. Such a pattern is typically seen in adult participants. Here we implemented a child-adapted version of the AB task used in the Ihssen and Keil (2009) study with adults. To this end, words were chosen to be age/grade appropriate and the number of lag conditions was reduced to shorten the experiment to an age-appropriate length. These adjustments aimed at ensuring that younger participants could read the words in the task with sufficient fluency, and that fatigue did not affect the results. Although the setup adjustments may not warrant a quantitative comparison, the similarities across tasks enable speculations regarding factors contributing to short-lived interference effects as found in the present research: when comparing overall accuracy patterns in our school-age students with those obtained from adult students (Ihssen and Keil, 2009), the younger group performed approximately 10% better for T2 identification at lag 2 and about 10% worse at the same lag for T1 than the older group. In the late temporal lags, similar levels (around 70%) of correct T2 report were noted in both samples. Although only suggestive at this point, this qualitative comparison gives rise to the conjecture that age-specific processing styles are contributing to the outcomes reported here. For instance, a processing style in which observers focus more strongly on the T2 than on the T1 stimulus would be expected to diminish the impact of affective content of T1. Age-related differences in strategy and AB performance have been discussed as a developing cognitive skill, allowing older individuals to shield T1 against subsequent distractors (Heim et al., 2011; Heim and Keil, 2012). The present study suggests that with this skill comes greater susceptibility for distraction effects when the protected T1 is distracting itself, e.g., by carrying emotional value. This speculative interpretation may lead to interesting questions to be tested in future research, such as how a certain processing strategy might be beneficial in one age cohort and unfavorable in another, or whether these styles are differentially related to higher-order cognitive skills and academic competency across youth. Alternatively, the confinement of emotional interference effects to the short lag interval may be taken to suggest that in young observers, emotional attention capture and AB interference reflect limitations in overlapping processes, not independent processes as seen in older adults. Again, this conjecture may be suitable for more rigid testing in work using robust electrophysiological markers of attentive processing.

In sum, the students' performance was characterized by enhanced report of pleasant T1 words, yet both pleasant and unpleasant T1s exerted pronounced interference on T2-word identification. These distraction effects were evident in the early temporal lag (i.e., during the critical AB interval), but did not extend into the late lag. Thus, our study demonstrates that in early adolescence affective information conveyed by words embedded in rapid visual streams obtains prioritized access to limited processing capacity, and thus interferes with subsequent target processing. As already stated in the Introduction, knowledge about the temporal limits of information processing is increasingly relevant, as hand-held devices, media exposure, and a rapidly expanding flow of information become more prevalent in the environment surrounding children and adolescents. In this context, the present findings may have implications for the design of electronic media employed in training and education. Such media could include tasks that promote attentional skills and learning in fast-paced arrays of relevant and irrelevant events; the use of certain perceptual strategies could then be shaped adaptively by instruction and feedback cues. Future work may further examine age-related differences in distractibility, implementing a wider spectrum of tasks and distractors to identify the constraints and benefits of sharing limited capacity between multiple sources of information.

### Conflict of interest statement

The authors declare that the research was conducted in the absence of any commercial or financial relationships that could be construed as a potential conflict of interest.

## Footnotes

^1^The target verbs on the test protocol were listed alphabetically rather than by affective category. Participants were informed a priori with respect to the alphabetic order but not about the varying degrees of pleasure. These provisions were implemented to control for a possible influence of a student's awareness of the word type on performance in the AB task.

## Appendix

**Table:**

**Pleasant first targets**	**Unpleasant first targets**
begeistern—to inspire^#^	drohen—to threaten^#^
belohnen—to reward	einsperren—to lock up
feiern—to party^#^	ekeln—to disgust^#^
freuen—to be glad^#^	erpressen—to blackmail
gewinnen—to win^#^	ersticken—to stifle
helfen—to help	foltern—to torture
jubeln—to cheer	lügen—to tell a lie^#^
küssen—to kiss^#^	morden—to murder
lachen—to laugh	quälen—to plague^#^
loben—to praise^#^	rauben—to rob
retten—to recover	schimpfen—to bluster
schenken—to make a gift^#^	schlagen—to bang^#^
siegen—to triumph	schmerzen—to pain
streicheln—to pet^#^	stehlen—to steal^#^
surfen—to surf	streiten—to fight
tanzen—to dance^#^	vergiften—to poison^#^
umarmen—to hug	verletzen—to injure^#^
verdienen—to earn	verprügeln—to batter^#^
verlieben—to fall in love^#^	würgen—to choke
wünschen—to wish	zerstören—to destroy^#^
**Neutral first targets**	**Neutral second targets**
befinden—to reside	aufstellen—to set up
benutzen—to use	behandeln—to treat
betreiben—to operate	betrachten—to look at
bilden—to form	beziehen—to upholster^#^
blicken—to gaze	binden—to tie
decken—to cover	drehen—to spin
einleiten—to introduce^#^	ergänzen—to complete^#^
einstellen—to adjust	erheben—to elevate
erwähnen—to mention^#^	festlegen—to determine
erweitern—to expand	gewöhnen—to get used to
flüstern—to whisper^#^	lauten—to read
holen—to fetch	lenken—to steer^#^
lehren—to teach	liefern—to deliver
messen—to measure	nennen—to name^#^
notieren—to take a note	nutzen—to make use
reichen—to last	rollen—to roll
sammeln—to collect^#^	schieben—to push^#^
sitzen—to sit	stammen—to stem from
verwenden—to utilize^#^	wenden—to turn
wechseln—to change	zählen—to count

Number sign denotes item was randomly selected for post-experimental affective word rating.
